# Clinical characteristics of patients assessed within an Improving Access to Psychological Therapies (IAPT) service: results from a naturalistic cohort study (Predicting Outcome Following Psychological Therapy; PROMPT)

**DOI:** 10.1186/s12888-016-0736-6

**Published:** 2016-02-27

**Authors:** Nilay Hepgul, Sinead King, Myanthi Amarasinghe, Gerome Breen, Nina Grant, Nick Grey, Matthew Hotopf, Paul Moran, Carmine M. Pariante, André Tylee, Janet Wingrove, Allan H. Young, Anthony J. Cleare

**Affiliations:** Department of Psychological Medicine & Centre for Affective Disorders, King’s College London, Institute of Psychiatry, Psychology & Neuroscience, London, UK; Department of Palliative Care and Rehabilitation, King’s College London, Cicely Saunders Institute, London, UK; King’s College London, Institute of Psychiatry, Psychology & Neuroscience, MRC Social, Genetic, and Developmental Psychiatry Centre, London, UK; Department of Psychology, King’s College London, Institute of Psychiatry, Psychology & Neuroscience, London, UK; South London & Maudsley NHS Foundation Trust, Centre for Anxiety Disorders and Trauma, London, UK; Department of Health Services and Population Research, King’s College London, Institute of Psychiatry, Psychology & Neuroscience, London, UK; South London & Maudsley NHS Foundation Trust, Southwark Psychological Therapies Service, London, UK

## Abstract

**Background:**

A substantial number of patients do not benefit from first line psychological therapies for the treatment of depression and anxiety. Currently, there are no clear predictors of treatment outcomes for these patients. The PROMPT project aims to establish an infrastructure platform for the identification of factors that predict outcomes following psychological treatment for depression and anxiety. Here we report on the first year of recruitment and describe the characteristics of our sample to date.

**Methods:**

One hundred and forty-seven patients awaiting treatment within an Improving Access to Psychological Therapies (IAPT) service were recruited between February 2014 and February 2015 (representing 48 % of those eligible). Baseline assessments were conducted to collect information on a variety of clinical, psychological and social variables including a diagnostic interview using the Mini International Neuropsychiatric Interview (MINI).

**Results:**

Our initial findings showed that over a third of our sample were not presenting to IAPT services for the first time, and 63 % had been allocated to receive higher intensity IAPT treatments. Approximately half (46 %) were taking prescribed psychotropic medication (most frequently antidepressants). Co-morbidity was common: 72 % of the sample met criteria for 2 or more current MINI diagnoses. Our initial data also indicated that 16 % met criteria for borderline personality disorder and 69 % were at high risk of personality disorder. Sixty-one percent scored above the screening threshold for bipolarity. Over half of participants (55 %) reported experiencing at least one stressful life event in the previous 12 months, whilst 67 % reported experiencing at least one form of childhood trauma.

**Conclusions:**

Our results to date highlight the complex nature of patients seen within an urban IAPT service, with high rates of psychiatric comorbidity, personality disorder, bipolarity and childhood trauma. Whilst there are significant challenges associated with researching IAPT populations, we have also confirmed the feasibility of undertaking such research.

## Background

The Improving Access to Psychological Therapies (IAPT) service was developed to provide psychological treatments for people with depression and anxiety in order to address both the high prevalence and burden of these disorders, and the number of untreated individuals [1, 2]. The therapeutic approaches used by IAPT are those recommended as first-line treatment for mild to moderate depression and anxiety by the National Institute for Clinical Excellence [3]. IAPT services offer both individual and group therapy options including cognitive behavioural therapy (CBT) and other NICE recommended talking therapies [4, 5]. The service is organised to provide stepped care, whereby patients are offered two levels of care (‘low intensity’ and ‘high intensity’) depending on their severity of symptoms and/or patient preference [6]. High intensity therapies include CBT, counselling and interpersonal psychotherapy and low intensity therapies include guided self-help, behavioural activation, mindfulness groups and wellbeing workshops. IAPT services use a standardised protocol and collect symptomatic and functional outcome data session-by-session. This provides an opportunity for researchers to study outcome data on large populations of individuals with common mental health disorders undergoing standardised, evidence based psychological treatments.

IAPT services now receive almost 900,000 referrals a year and more than half of those referred successfully enter treatment [7]. Over its first 3 years, the IAPT programme reported early successes, notably the treatment of “the first million patients” [5]. Overall recovery rates were 45 % in the last quarter of 2011/12, demonstrating consistent improvement over the duration of the programme [5]. Recovery rates are defined as moving from caseness to non-caseness on self-rated measures of low mood and anxiety. A score of 10 or more on the Patient Health Questionnaire (PHQ-9) is used to indicate caseness for depression and a score of 8 or more on the Generalized Anxiety Disorder 7 (GAD-7) is used to indicate caseness for anxiety [8, 9]. Whilst these recovery rates are encouraging, they also indicate that approximately half of patients are not meeting standard definitions of recovery at the end of their treatments. Furthermore, it is likely that a substantial proportion of those who do recover may go on to relapse in due course. Our knowledge of predictors of treatment response for depression and anxiety, both in terms of psychological and pharmacological treatments, is limited. It is likely that depression and anxiety have many underlying causes, across psychological, social, and biological domains, all of which may feasibly affect outcome or choice of treatment. Only by studying large cohorts of patients receiving treatments, will it be possible to identify factors which predict positive or negative response. Indeed, by furthering our understanding of this, new treatment targets can be developed, and existing treatments can be more effectively applied.

The predicting outcome following psychological therapy in IAPT (PROMPT) project provides an infrastructure to allow for the systematic collection of data geared towards understanding the predictors of treatment outcomes. Furthermore, through PROMPT we can identify subgroups of participants who do not respond to existing treatments in order to devise experimental studies for the identification of new treatments (both psychological and pharmacological). To our knowledge, this is the first study to collect systematically both clinical and biological data within an IAPT population and we have previously published the complete protocol for this project [6]. The main objective of the PROMPT project is to identify factors that predict response or lack of response to psychological treatment delivered by IAPT services for depression and anxiety. Here, we report on the sample from the first year of recruitment. Our main aim in this report was to describe in detail the demographic and clinical characteristics of the sample recruited to date, including potential implications for IAPT services and the representativeness of the study. All biological samples will be analysed in relation to treatment outcome and therefore are not available for reporting at this early stage.

## Methods

### Study design and participants

This project uses a naturalistic, observational design. All patients are recruited from one South London IAPT service – Southwark Psychological Therapies Service (SPTS). All eligible patients referred (self-referral or via general practitioner) to SPTS are initially asked to consent to be contacted for research purposes as part of standard clinical practice. Patients who consent for research contact are identified via the IAPT electronic patient record system (IAPTus). Identified patients are then approached by post, telephone or email and asked to take part in the project prior to starting their therapy. Inclusion criteria for this project are any patients who are accepted by the IAPT service for treatment and are able to give informed consent. Patients are excluded if they are not sufficiently fluent in English (as indicated on their electronic record by the requirement of an interpreter). Written informed consent is obtained from all participants after a complete explanation of the study, a presentation of a participant information sheet and an opportunity to ask questions. All data are collected at a baseline research visit which takes place at the NIHR/Wellcome Trust King’s Clinical Research Facility. This visit involves a diagnostic interview carried out with a trained researcher, completion of a range of questionnaires and collection of biological samples (blood and hair). All participants included in this report were recruited between February 2014 and February 2015. The project was approved by the Bromley NHS Research Ethics Committee (Ref: 13/LO/1347).

### Demographic and treatment factors

Date of birth and self-identified ethnicity was collected from the participants’ electronic IAPT patient record. Information regarding treatment such as the number of previous IAPT episodes and whether or not the participant is due to receive high intensity or low intensity IAPT therapy was also collected from these electronic records. High intensity therapies include individual CBT, counselling and interpersonal psychotherapy. Low intensity therapies include guided self-help, behavioural activation, mindfulness groups and wellbeing workshops. Additional demographic information was collected as part of the questionnaire measures participants are administered during the PROMPT assessment including: relationship status, educational attainment, employment status, housing status and household income.

### Diagnoses & symptomatology

The Mini International Neuropsychiatric Interview (MINI) was used to assess current and lifetime diagnoses for all participants. The MINI is a structured interview which assesses DSM diagnoses, and is rapid to administer. The MINI covers the following diagnoses: major depressive episode (including recurrent major depression and major depression with melancholic features); dysthymia; suicidality; mania and hypomania; panic disorder; social phobia; agoraphobia; obsessive compulsive disorder; post-traumatic stress disorder; alcohol abuse; alcohol dependence; substance abuse; substance dependence; psychotic disorders; mood disorder with psychotic features; anorexia; bulimia; generalised anxiety disorder; and antisocial personality disorder [10]. During the interview participants were also administered the borderline personality subsection of the Structured Clinical Interview for DSM-IV Personality Disorders (SCID-II) and the Hypomania Checklist (HCL-16) [11, 12]. The HCL-16 is a 16 item measure where a score of ≥8 is used as a cut-off point suggestive of some degree of bipolarity.

In addition to the diagnoses established by the MINI, depression and anxiety symptoms were further assessed using the Patient Health Questionnaire (PHQ-9) and the Generalized Anxiety Disorder assessment (GAD-7) [8, 9]. Both of these measures are self-report and are routinely collected as part of standard IAPT practice at every treatment session which will allow us to compare pre- and post-therapy scores. A score of ≥10 on the PHQ-9 is used to indicate caseness. For use as a Generalized Anxiety Disorder (GAD) screen, a score of ≥10 is also recommended on the GAD-7. However, it also has satisfactory (albeit lower) sensitivity and specificity for detecting other anxiety disorders when a cut off of ≥8 is used and indeed this is the cut-off used by IAPT services to indicate caseness. The Standardised Assessment of Personality Abbreviated Scale (SAPAS) was used to screen for personality disorder [13]. This is an eight item measure where a score of ≥3 is indicative of cases at high risk of personality disorder.

### Psychosocial stress

Stressful life events were assessed using the List of Threatening Events Questionnaire [14]. This is a self-report questionnaire examining the incidence of 12 categories of negative life events involving moderate or long-term threats such as illness or injury, the death of a close friend or relative, unemployment, financial loss and loss of important relationships. Participants indicate whether they have experienced such an event and the date that it occurred. We focused specifically on life events which took place in the 12 months prior to the baseline interview in order to have an indication of recent stressors prior to engaging in IAPT services. A dichotomised variable was created to indicate: no life events experienced in the previous 12 months, versus one or more life events experienced in the previous 12 months.

Traumatic events during childhood were assessed using the Childhood Trauma Questionnaire [15]. This is a self-report measure that assesses five domains of trauma occurring prior to age 17. These are: emotional abuse, physical abuse, sexual abuse, emotional neglect, and physical neglect. Each item is rated on a five point Likert scale from “never true” to “very often true”, with scores for each sub scale ranging from 5 to 25. The authors provide the following severity indications: emotional abuse (5–8, none; 9–12, low; 13–15, moderate; ≥16 severe); physical abuse (5–7, none; 8–9, low; 10–12, moderate; ≥13 severe); sexual abuse (5, none; 6–7, low; 8–12, moderate; ≥13 severe); emotional neglect (5–9, none; 10–14, low; 15–17, moderate; ≥18 severe); and physical neglect (5–7, none; 8–9, low; 10–12, moderate; ≥13 severe).

### Data analyses

All data were analyzed using IBM SPSS statistical software version 20. Continuous variables are presented as mean ± SEM.

## Results

### Recruitment

**Fig. 1:**
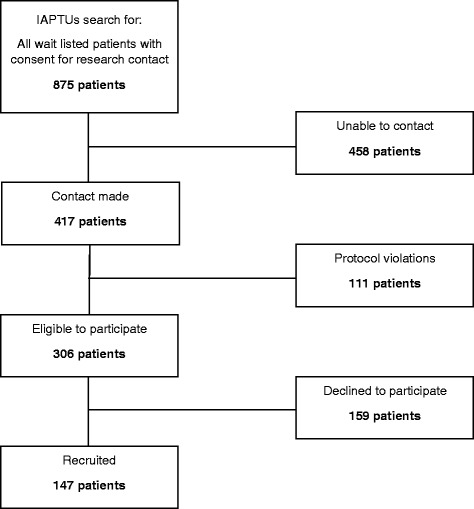
Recruitment flow

One hundred and forty-seven patients were successfully recruited during the first year of the PROMPT project. Figure 1 illustrates the recruitment flow. We were unable to make full contact with 50 % of the patients identified by our searches. Participants were deemed to be uncontactable once all contact methods had been explored (letter in the post, minimum of two phone call attempts and an email). Once contact with potential participants had been established, their eligibility was re-assessed and those who violated the protocol were excluded from participating. Protocol violations included those who had already begun their therapy at the point of contact or those who indicated they would no longer be receiving therapy within Southwark Psychological Therapies Service. As such, 306 patients were deemed to be eligible to participate in the study and 48 % of these patients were successfully recruited. The foremost reason for patients declining to participate was due to not having time to be able to attend the research interview prior to starting therapy.

### Demographic and treatment factors

The demographic characteristics of the sample are presented in Table 1. Our sample was predominantly comprised of patients who were waiting to receive higher intensity IAPT treatments (63 %) most often individual CBT. For 38 % of the sample, this was not the first IAPT episode and approximately half (46 %) were taking prescribed psychotropic medication at the time of the interview (most frequently antidepressants: 28 patients were taking citalopram, 17 patients were taking sertraline, seven patients were taking fluoxetine, five patients were taking mirtazapine, four patients were taking amitriptyline and three patients were taking paroxetine). Duration of current illness was available in 85 participants and the median was 241 days.

**Table 1:** Socio-demographic characteristics of the sample

Age (years)	
Mean ± SEM	40.3 ± 1.1
Range	18–77
Gender	
Females	94 (64 %)
Males	53 (36 %)
Ethnicity	
White British	90 (61 %)
White Other	32 (22 %)
Mixed	5 (3 %)
Black	6 (4 %)
Pakistani/Indian/Bangladeshi	4 (3 %)
Other	10 (7 %)
Education Level^a^	
No Qualifications	15 (11 %)
GCSEs/O Levels/NVQ	22 (15 %)
A Levels/GNVQ	36 (25 %)
Higher degree	71 (49 %)
Employment^a^	
Full-time	60 (41 %)
Part-time	18 (13 %)
Student	15 (10 %)
Unemployed	29 (20 %)
Sick Leave/Homemaker	23 (16 %)
Relationship Status^a^	
Single	62 (44 %)
Steady relationship	34 (24 %)
Married	37 (26 %)
Separated/Divorced	7 (5 %)
Widowed	2 (1 %)
Housing^a^	
Owned/Mortgaged	37 (26 %)
Rented private	41 (28 %)
Rented from local authority	52 (36 %)
Other	14 (10 %)
Household income^a^	
£0–£5,475	31 (22 %)
£5,476–£12,097	15 (11 %)
£12,098–£20,753	19 (13 %)
£20,754–£31,494	26 (18 %)
Above £31,495	52 (36 %)

^a^Missing variables (*n* = 2–5)

### Diagnoses and symptomatology

**Fig. 2:**
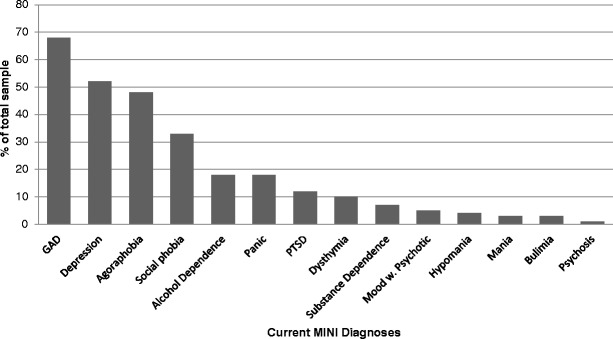
Prevalence rates for current MINI diagnoses

**Fig. 3:**
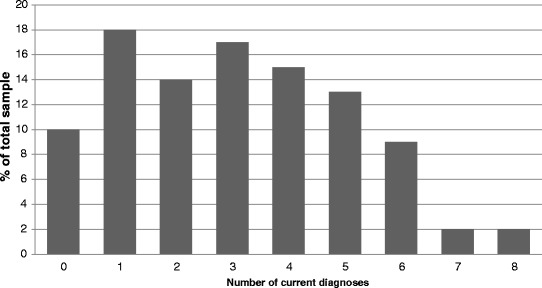
Prevalence rates for multiple MINI diagnoses

The MINI allows for the identification of both current and lifetime diagnoses. Table 2 illustrates the prevalence of all MINI diagnoses in our sample. We specifically investigated the current diagnoses as well as the number of multiple diagnoses given; these are presented in Figs. 2 and 3 respectively. Seventy-two percent of the sample met criteria for 2 or more current MINI diagnoses. Further to the MINI diagnoses, our initial data also indicates 16 % of the sample met criteria for borderline personality disorder and 69 % scored above the cut-off (≥3) on the personality disorder screen. Moreover, 61 % scored above the cut-off (≥8) for hypomania on the HCL-16. Finally, the mean depression and anxiety scores of the sample as measured by the PHQ-9 and GAD-7 were 13.4 ± 0.6 and 12.0 ± 0.5 respectively. Seventy-two percent of the sample scored above the cut-off (≥10) for depression and 78 % scored above the cut-off (≥8) used by IAPT services for anxiety. The complete breakdown of the severity of PHQ-9 and GAD-7 scores are presented in Table 3.

**Table 2:** Prevalence rates for all MINI diagnoses

Diagnosis	*n* (% of total sample)
Depression	
Current	77 (52 %)
Recurrent	53 (36 %)
Dysthymia	14 (10 %)
Suicidality	
Low	57 (39 %)
Moderate	14 (10 %)
High	5 (3 %)
Hypomania	
Current	6 (4 %)
Past	24 (16 %)
Mania	
Current	4 (3 %)
Past	18 (12 %)
Panic Disorder	
Current	26 (18 %)
Lifetime	37 (25 %)
Agoraphobia	70 (48 %)
Social Phobia	
Generalized	33 (22 %)
Non-generalized	16 (11 %)
Obsessive Compulsive Disorder	27 (18 %)
Post-Traumatic Stress Disorder	18 (21 %)
Alcohol Dependence	27 (18 %)
Substance Dependence	11 (7 %)
Mood disorder with Psychotic features	
Current	7 (5 %)
Lifetime	10 (7 %)
Psychosis	
Current	2 (1 %)
Lifetime	5 (3 %)
Bulimia	5 (3 %)
Generalized Anxiety Disorder	100 (66 %)
Antisocial Personality Disorder	6 (4 %)

**Table 3:** Breakdown of PHQ-9 and GAD-7 scores

	*n* (% of total sample)
PHQ-9^a^	
Minimal (0–4)	17 (12 %)
Mild (5–9)	23 (16 %)
Moderate (10–14)	40 (28 %)
Severe (≥15)	64 (44 %)
GAD-7^a^	
Minimal (0–4)	16 (11 %)
Mild (5–9)	30 (21 %)
Moderate (10–14)	46 (32 %)
Severe (≥15)	52 (36 %)

^a^Missing variables (*n* = 3)

### Psychosocial stress

Firstly, we investigated the prevalence of stressful live events in the previous 12 months in our sample. Fifty-five percent of participants reported experiencing at least one life event in the previous 12 months. We also investigated the prevalence of childhood trauma. Just under a third (29 %) of participants reported moderate or severe emotional abuse, 16 % reported moderate or severe physical abuse, 21 % reported moderate or severe sexual abuse, 31 % reported moderate or severe emotional neglect and 23 % reported moderate or severe physical neglect. Sixty-seven percent of participants reported experiencing at least one form of childhood trauma. The complete breakdown of the types and severity of childhood trauma are presented in Table 4.

**Table 4:** Prevalence rates of childhood trauma

Trauma Type	*n* (% of total sample)
Emotional Abuse^a^	
None	74 (52 %)
Low	26 (19 %)
Moderate	11 (8 %)
Severe	30 (21 %)
Physical Abuse^a^	
None	114 (80 %)
Low	6 (4 %)
Moderate	10 (7 %)
Severe	12 (9 %)
Sexual Abuse^a^	
None	92 (65 %)
Low	20 (14 %)
Moderate	18 (13 %)
Severe	11 (8 %)
Emotional Neglect^a^	
None	61 (43 %)
Low	37 (26 %)
Moderate	17 (12 %)
Severe	28 (19 %)
Physical Neglect^a^	
None	91 (64 %)
Low	18 (13 %)
Moderate	15 (10 %)
Severe	19 (13 %)

^a^Missing variables (*n* = 4–6)

## Discussion

To our knowledge, this is the first study to systematically assess the diagnostic case-mix of an Improving Access to Psychological Therapies (IAPT) service. Much of the previous data on IAPT services comes from IAPT reports or only includes data collected as part of standard clinical practice. The PROMPT protocol provides an opportunity for the systematic collection of additional and detailed clinical, psychosocial and biological data. Here, we have presented the progress of the study in its first year and a description of the sample. Our early findings have highlighted two key points: the complexities present within an ostensibly straightforward population with common mental disorders treated at the primary care/secondary care interface, and also the challenges of recruiting patients from routine clinical care into a naturalistic study such as this.

Our initial data show the degree of psychiatric comorbidity present in patients seen within the Southwark IAPT service. Based on the diagnoses obtained using the MINI, we have shown that comorbidity is the rule: 14 % of our sample met criteria for two current diagnoses and the majority (58 %) had three or more current diagnoses. This degree of comorbidity is in keeping with the figures reported by Southwark Psychological Therapies Service where 53 % of all patients in the service were found to meet criteria for two or more diagnoses on the Psychiatric Diagnostic Screening Questionnaire (PDSQ) [16]. Similarly, the proportion of caseness identified using the PHQ-9 and GAD-7 in our sample are also consistent with those reported by the service (72 and 78 % versus 71 and 74 %, respectively). Other national reports of caseness within IAPT services as identified using the PHQ-9 and GAD-7 are also similar at 84 and 79 % in one study [17], and 70 and 68 % in another [18]. Taken together, these data indicate that our sample is clinically representative of that seen by the IAPT service as a whole both locally and nationally. We also found high rates of likely traits of personality disorder, with structured interviewing suggesting the definitive presence of borderline personality disorder in 16 % and more general screening suggesting around two-thirds had some features of personality disorder. It has recently been demonstrated that the presence of co-morbid personality difficulties adversely affects treatment outcome among individuals attending IAPT treatment [19]. In addition to the level of comorbidity, for over a third of our sample, this was not the first presentation to the Southwark IAPT service.

Our results also indicate a high level of potential bipolarity in this population. As well as 28 % with a lifetime history of mania or hypomania, we found that 61 % of participants scored above the cut-off on the HCL-16, suggesting a large proportion of the patients with depression seen within the IAPT service fall within the “soft” bipolar spectrum [20, 21]. This is in keeping with data from the large Bipolar Disorder: Improving Diagnosis Guidance and Education (BRIDGE) study where a prevalence rate of 58.7 % was found using the longer 32 item hypomania checklist (HCL-32) in a population of community and hospital patients with depression [22]. Unrecognised bipolarity is thought to be a significant factor contributing to treatment resistance in depression [23], and is therefore of great potential significance as a possible predictor within the current study. Furthermore, the management of bipolar spectrum disorders is complex and differs from that of unipolar depression, both in terms of pharmacological and psychological therapy with particular uncertainty about the benefits or otherwise of antidepressants [24]. Also, and of specific relevance for IAPT services, there is less evidence from clinical trials for the use of psychological therapies such as CBT in bipolar spectrum disorders, and results have been inconclusive [25–27]. The focus of such therapies may also need to be different in the presence of a bipolar diathesis. Recurrence rates are also higher: approximately 60 % of patients with bipolar disorder relapse within 2 years of remission from a major depressive or manic episode [28].

Perhaps in keeping with the diagnostic complexities, our initial data also provides evidence for a high prevalence of childhood trauma in this population. This is not surprising as the association between childhood trauma and increased risk for adult psychopathology (especially depression) is well documented [29–31]. Moreover, a history of childhood trauma has also been shown to be associated with other disorders including bipolar disorder and personality disorder [32–34], both of which are prevalent in our sample. In relation to treatment outcomes, a recent meta-analysis in depression revealed that maltreated individuals were twice as likely to have a poorer prognosis when compared to those without any history of childhood trauma [35]. Indeed, this high prevalence of childhood trauma in combination with the diagnostic complexities may contribute to the low recovery rates seen in this population. Furthermore, we also demonstrate a substantial prevalence of alcohol and drug dependence in our sample (18 and 7 %, respectively) both of which may also have serious implications for treatment outcomes.

Taken together, our early findings suggest that patients seen by Southwark IAPT have complex psychopathologies. Given that IAPT services were originally targeted for individuals with mild to moderate depression and anxiety, and are often seen as an early intervention/primary care service, this high level of multi-morbidity may pose significant implications for treatment provision and outcomes. The presence of psychiatric comorbidities may affect treatment outcomes in a number of ways such as increased rates of treatment drop out, and are a recognized factor conferring a worsened outcome to treatment in general. It may also lead to difficulties in establishing effective therapeutic relationships and therefore require additional training for IAPT staff. There is existing evidence to suggest that patients with complex psychopathologies might benefit from alternative therapies or integrative therapies. Integrative therapies can allow clinicians to combine interventions so that they are tailored for the presence of comorbidity. This has been suggested to be useful for the treatment of comorbid personality disorder [36, 37] and for generalized anxiety disorder [38]. However, this may require additional training for IAPT staff and have associated cost implications. Our findings suggest that large numbers of patients presenting to these services may have significant needs over and above those likely to be met by the relatively brief interventions that IAPT services are currently expected and are able to provide.

Collecting data from participants in a naturalistic setting, whilst providing ecologically valid data, is not without its limitations. This is a naturalistic, observational project and therefore our original sample size estimates were based on patient throughput and human resources. We had estimated to recruit up to 600 patients in the first year of the project [6]. However, our results from the first year of recruitment have highlighted appreciable difficulties in the process of recruitment. The first main obstacle is that only a minority of patients attending Southwark IAPT consent to be contacted for any research purposes. A second major obstacle has been that we were unable to establish contact with 50 % of the identified potential participants. Additionally, 63 % of the participants recruited in to the study were waiting to receive high intensity treatments. This is a higher proportion than what might be expected of an IAPT service where usually low intensity treatments are more frequent. An evaluation of five primary care trusts reported that 57.3 % of patient received low intensity treatments and 26.2 % received high intensity [18]. One explanation for this difference is that high intensity treatments involve a substantial waiting period whereas low intensity treatments are started much more quickly. As such, there is a smaller window of opportunity to contact patients and involve them in research prior to starting therapy. Thus, an important outcome from the study to date has been identifying these areas as potential factors to be considered both in the ongoing implementation of the current study, but also in the design and recruitment processes of any similar studies in future. This may require organisational changes in order to better embed research in to clinical practice.

There are some limitations to this study. Firstly, the PROMPT project focusses on one urban London IAPT service (Southwark). As such; the degree to which the figures presented may generalize to other IAPT services, including those in more rural areas, needs further exploration. Secondly, the participants are recruited via the consent for contact initiative and it is possible that those who agree to research contact are not representative of all patients referred to and seen in Southwark IAPT. This would be of particular concern if increased likelihood of participation in research was perhaps related to comorbidity. However, epidemiological evidence suggests that more severe psychopathology is associated with a reduced likelihood of participation in research [39] and hence it is probable that the patients who declined to participate had more, not less, psychopathology. Furthermore, it is unlikely that our sample is clinically more severe as comparisons with figures reported by Southwark Psychological Therapies Service confirm that our sample is clinically and demographically similar to the population of the service [16]. In terms of study methodology, the main limitations are those of the underlying tools used. Thus, for example, the estimates of bipolarity are limited by the uncertain nature of a retrospective assessment of hypomania, and by the sensitivity/specificity of the HCL questionnaire. The estimates of personality disorder traits are similarly limited by the self-report nature of the tools used, and the likely overestimate of such traits when assessed during a depressive episode. Nevertheless, we believe that the findings are valid within these constraints, and within the limits of the information that can realistically be obtained from a sample of patients such as this.

## Conclusions

In conclusion, the results to date from the PROMPT project confirm the feasibility of such a study, whilst emphasizing the very significant challenges that are faced when recruiting in this population. Moreover, the results have revealed the complex nature of the patients seen within an urban IAPT service, with high rates of psychiatric comorbidity, bipolarity, childhood trauma and traits of personality disorder.

## Abbreviations

BRIDGE: bipolar disorder: improving diagnosis guidance and education
CBT: cognitive behavioural therapy
DSM: diagnostic and statistical manual of mental disorders
GAD: generalized anxiety disorder
GAD-7: generalized anxiety disorder (7 item)
HCL-16: hypomania checklist (16 item)
HCL-32: hypomania checklist (32 item)
IAPT: improving access to psychological therapies
IAPTus: improving access to psychological therapies patient management software
MINI: mini international neuropsychiatric interview
NICE: national Institute for Health and Care Excellence
NIHR: National Institute for Health Research
PDSQ: Psychiatric Diagnostic Screening Questionnaire
PHQ-9: patient health questionnaire (9 item)
PROMPT: predicting outcome following psychological therapy in IAPT
PTSD: post traumatic stress disorder
SAPAS: standardised assessment of personality abbreviated scale
SCID-II: structured clinical interview for DSM-IV axis II personality disorders
SEM: standard error of the mean
SPTS: southwark psychological therapies service
